# Characteristics and 12-month outcomes of clinically-referred children and young people at risk of body dysmorphic disorder

**DOI:** 10.1007/s00127-025-03023-x

**Published:** 2025-12-22

**Authors:** Eleni Frisira, Sue Fen Tan, Chris Partlett, Grace Holt, Georgina Krebs, Argyris Stringaris, Tamsin Marshall, Kapil Sayal

**Affiliations:** 1 Institute of Mental Health, School of Medicine, Mental Health and Clinical Neurosciences, University of Nottingham, Nottingham, NG7 2TU UK; 2 Nottingham Clinical Trials Unit, University of Nottingham, Applied Health Research, Building 42, NG7 2RD Nottingham, UK; 3 Research Department of Clinical, Educational and Health Psychology, 1-19 Torrington Place, University College London, London, WC1E 7HB UK; 4 Faculty of Brain Sciences, University College London, London, UK; 5 National and Kapodistrian University of Athens, Athens, Greece; 6 Berkshire Healthcare NHS Foundation Trust, Bracknell, UK

**Keywords:** Body dysmorphic disorder, Adolescence, CAMHS, DAWBA, STADIA

## Abstract

**Purpose:**

Body Dysmorphic Disorder (BDD) is impairing yet often under-detected in children and young people (CYP). This paper aims to describe the characteristics and 12-month outcomes of clinically referred CYP based on their likelihood of having BDD.

**Methods:**

This paper investigated 307 CYP aged 11–17 years from the STandardised DIagnostic Assessment (STADIA) trial for CYP with emotional difficulties referred to Child and Adolescent Mental Health Services (CAMHS). CYP and/or their parents completed the Development and Wellbeing Assessment (DAWBA) to assess the likelihood of meeting diagnostic criteria for emotional disorders, including BDD. Baseline and 12-month follow-up measures were collected from self- and/or parent- reports and healthcare records.

**Results:**

Overall, 6.2% (95% confidence interval 3.8%, 9.5%) of referred CYP had probable BDD according to the DAWBA; however, no CYP received a BDD diagnosis from CAMHS clinicians. CYP with probable BDD were predominantly female (84%), white (89%) and came from diverse socio-economic backgrounds. Nearly three quarters (74%) of them scored very high for comorbid depression and generalised anxiety disorder, but only 47% had their referral accepted by CAMHS by 12 months. At baseline, 50% reported self-harm thoughts and 33% reported self-harm behaviours, with 42% reporting self-harm behaviours at 12 months.

**Conclusions:**

BDD is relatively common among CYP with emotional difficulties referred to CAMHS, but may be overlooked. CYP with probable BDD often meet criteria for depressive or anxiety disorders and exhibit high levels of self-harm thoughts and behaviours. Despite this, referral acceptance to CAMHS was no higher for CYP with probable BDD, highlighting the need to raise awareness about BDD among referrers and clinicians.

**Supplementary Information:**

The online version contains supplementary material available at https://doi.org/10.1007/s00127-025-03023-x.

## Introduction

Body dysmorphic disorder (BDD) is characterised by excessive preoccupations with perceived defects or flaws in appearance leading to significant levels of distress and functional impairment [1, 2] Affected individuals engage in time-consuming, repetitive behaviours or mental acts in response to their appearance preoccupations [2–4]. While symptom onset most commonly occurs around 12–13 years of age, a clinical diagnosis, for those who receive one, is often delayed until around 16 years of age, suggesting that many cases may go unrecognised for years [5–7].

BDD is frequently under-detected due to young people’s reluctance to seek mental health care, limited clinician awareness and attribution of appearance preoccupation symptoms to typical adolescents’ concerns [8–10]. Adolescent BDD is also associated with increased risk of self-harm and up to 3.8 times greater risk of suicidal ideation [6, 11, 12]. There is also an increased lifetime risk of attempted suicide, with the risk of completed suicide amongst individuals with BDD being 3.5 times higher than those without it [13]. Without effective intervention, BDD often remains a chronic condition, has high psychiatric comorbidity including obsessive compulsive disorder (OCD), mood and anxiety disorders, and results in psychosocial difficulties including poor school attendance [14–16].

In the UK, around 1% of 5–19 year-olds in the general population are estimated to have BDD, with a higher prevalence among girls (1.8%) than boys (0.3%) [17, 18]. The prevalence of BDD increases with age, going from 0.1% at ages 5–10, to 1% at ages 11–16, and reaching 3.1% at age 17–19 [17]. However, much less is known about BDD in clinically referred populations. To our knowledge, no international studies have focused on BDD in general Child and Adolescent Mental Health Service (CAMHS) referred samples. In the UK, CAMHS is a multi-disciplinary specialist mental health service that supports children and young people (CYP) with mental health difficulties, up to the age of 18 years [19]. It forms part of the National Health Service (NHS) and CYP are usually referred by health, social care or education professionals, when their difficulties require specialist assessment and intervention beyond what can be provided in primary care [20]. It might be expected that the prevalence of mental health conditions, including BDD, is higher in populations referred to specialist services like CAMHS compared to the general population [21]. One UK study set in specialist outpatient CAMHS OCD and mood disorder clinics found that the prevalence of receiving a clinical diagnosis of BDD in CYP was around 22–24% [22].

BDD is common and impairing, and there is an urgent need to better understand how affected young people present in order to improve identification and outcomes in routine clinical practice [22–24]. Early identification is important to signpost young people to appropriate services in a timely manner, and to prevent worsening of symptoms and the development of comorbid mental health disorders [19]. Therefore, this study aims to describe: (a) the baseline characteristics, including sociodemographic factors and other psychopathology; and (b) the 12-month outcomes, including service-related outcomes and changes in psychopathology, of clinically referred 11–17 year-olds based on their likelihood of having BDD.

## Methods

### Sample

STADIA (STandardised DIagnostic Assessment for children and young people with emotional difficulties) is a multi-centre randomised controlled trial investigating the clinical and cost effectiveness of a standardised diagnostic assessment tool (the Development and Wellbeing Assessment; DAWBA) [25] in CAMHS. The intervention arm involved completion of the DAWBA at the point of referral receipt into CAMHS in addition to the assessment as usual, and the comparator arm was assessment as usual [25]. The STADIA trial involved (1) CYP aged 5–17 years with emotional difficulties, who were referred to outpatient CAMHS around sites in England, and (2) their parent (or carer). Participants in the underlying trial were included if their referral mentioned any current emotional difficulties, regardless of the number, frequency, or severity of these difficulties [25]. “Worries/concerns about appearance” were among the keywords used to screen referrals [25]. Urgent referrals were excluded, to allow enough time for the DAWBA to be completed before offering clinical input [25]. The sample for this paper reflects 307 CYP aged 11–17 years that had the DAWBA section on “Worrying about Physical Appearance” completed by their parent and/or themselves. A diagram illustrating the selection process for the sample included in this analysis from the STADIA trial is provided in Online Resource 1.

For CYP aged 11–15 years, the parent served as the primary participant, with the option for the young person (secondary participant) to contribute data with their parent’s consent. In contrast, young people aged 16–17 years were the primary participants, with the option for their parent (secondary participant) to contribute data with the young person’s consent. STADIA participants below the age of 11 were excluded from this analysis, due to parents only completing the DAWBA and BDD being rare in this age group [10]. Further information on the full STADIA trial details available via the published protocol [25]. Ethics Committee approval: South Birmingham Research Ethics Committee (Ref. 19/WM/0133).

### Measures

#### DAWBA

The DAWBA is a set of structured questionnaires/interviews designed to measure the likelihood of common emotional and behavioural disorders in CYP [26]. Questions are based on diagnostic criteria as operationalised in ICD-10, DSM-IV, and most recently DSM-5 [26]. The DAWBA encompasses different sections covering individual disorders, including a section focusing on appearance pre-occupation that is related to BDD [26]. Each section opens with a screening question(s), which determines whether participants will be asked to complete the full section. After the initial screening item for BDD on worrying about physical appearance, subsequent questions cover preoccupation with distinct features (such as skin, hair, facial features and other body parts), behavioural compulsions (repeatedly comparing, checking, hiding, and seeking ways to improve appearance), associated distress, and impact on social, family and school life [18, 25]. A fully structured, self-respondent DAWBA questionnaire was used for CYP and/or their parents, excluding open-ended questions [25]. The specific items included in the BDD section have been described in detail in previous epidemiological research [18] and further information on the DAWBA is available at http://www.dawba.info.

#### The DAWBA algorithm

The DAWBA collects information from different informants (CYP and/or parent) to then assign CYP to a likelihood of meeting the criteria for a disorder [27]. This reflects a likelihood prediction rather than an actual clinical diagnosis and corresponds to approximate epidemiological prevalence in community samples [27]. The DAWBA algorithm has been validated for a number of diagnoses in CYP, including separation anxiety, specific phobia, social phobia, panic and agoraphobia, generalised anxiety, post-traumatic stress disorder, OCD, depression, oppositional defiant disorder and conduct disorder [27]. It has been shown to have a sensitivity and specificity of 83.8% and 81.5% respectively for detecting BDD [22]. Further details on the DAWBA algorithm for the “worrying about physical appearance” section, including distribution of symptom and impact ratings by CYP and/or parents, are provided in Online Resource 2.

#### The DAWBA categories

In this study, the likelihood of meeting diagnostic criteria for BDD was determined using the algorithm-generated diagnostic prediction based on DAWBA symptom and impact responses as reported by CYP and/or their parents (Online Resource 2) [22]. For cases where the DAWBA was only completed by either the CYP or the parent, single informant ratings were used. Participants were then divided in the following categories for likelihood of BDD.

- Probable: Both CYP and parent reported high symptom and impact ratings.
- Possible: One informant (either CYP or parent) reported high symptom and impact ratings, while the other informant reported medium or low symptom ratings or did not provide a response.
- Unlikely: Either CYP or parent, or both, reported medium or low symptom and impact ratings. Participants answering ‘no’ to the screening item of the BDD section of the DAWBA were included in this category.

#### Other baseline measures

The analysis incorporated several baseline measures at the point of referral to CAMHS and inclusion to the study. These measures were collected from healthcare records and parent/self-reports and included:

- CYP demographic data: age, sex, ethnicity;
- socioeconomic status: the index of Multiple Deprivation score derived from the postcode of the CYP’s residence;
- previous referral to CAMHS;
- algorithm-derived diagnostic prediction highlighting a very high likelihood of a CYP meeting the international classification criteria for one of the following emotional disorders based on DAWBA responses. The DAWBA domains assessed corresponded to the following emotional disorders: separation anxiety, specific phobia, social phobia, panic and agoraphobia, generalised anxiety, post-traumatic stress disorder, obsessive-compulsive disorder, depression.

#### Outcome measures

Outcome measures were collected at 12 months follow-up from healthcare records, and self- and/or parent- reports. During the 12-month follow-up period, participants’ DAWBA reports were uploaded to their CAMHS clinical record for clinicians to access as an adjunct to treatment as usual [25].

- Referral acceptance by CAMHS: defined as the CYP being offered an initial CAMHS appointment.
  i. Acceptance of index referral (the referral at the point of STADIA trial recruitment).
  ii. Acceptance of any CAMHS referral (index or other) within 12 months.
- Clinician-made, recorded diagnosis of an emotional disorder within 12 months.
- Treatment/Intervention offered by CAMHS within 12 months (yes/no; irrespective of whether this was received).
- Treatment/Intervention started by CAMHS within 12 months (yes/no).
- Revised CYP Anxiety and Depression Scale (RCADS) completed by CYP and/or parents. RCADS is a 47-item self-reported measure of high validity, with each item rated on a 4-point scale and total score of up to 141. Higher scores are associated with greater symptom severity [28].
- CYP self-report of self-harm (i) thoughts three or more times, and (ii) behaviour two or more times in the last 6 months.

### Analysis

Descriptive statistics were used to compare the baseline and outcome measures across the groups. Continuous scores were summarised in terms of the mean and standard deviation (SD) in each of the groups. Categorical data were summarised in terms of frequency counts and percentages in each of the groups. The proportion of participants in each of the BDD likelihood categories is presented alongside 95% confidence intervals (CI). No formal statistical comparisons were made because of the large number of comparisons and the exploratory nature of the analyses. Therefore, all analyses are descriptive and intended to characterise the sample rather than draw inferences. Differences between groups are described in terms of magnitude; small differences (< 5%) are interpreted as similar, whereas larger differences (5% or more) are described as ‘higher’ or ‘lower.’

## Results

### BDD likelihood prediction

Nineteen CYP (6.2% (95% CI 3.8%, 9.5%)) were identified through the DAWBA as having probable BDD, 95 (30.9% (95% CI 25.8%, 36.4%)) as possible BDD and 193 (62.9% (95% CI 57.2%, 68.3%)) as unlikely to have BDD (Table 1). Amongst those with a very high likelihood of meeting diagnostic criteria for an emotional disorder other than BDD (according to the DAWBA), 8.5% (95% CI 5.2%, 13.0%) had probable BDD. From the total 307 CYP included in the sample (i.e. where either the CYP and/or parent had completed the DAWBA), 15.3% of parent reports and 50.4% of CYP reports were missing due to the nature of the study design of the underlying trial (Table 1). Specifically, in the 11–15 year age group, 17.6% of parents reported high DAWBA ratings where young people’s responses were missing. Among those aged 16–17 years, 34.9% of young people reported high DAWBA ratings, while their parent ratings were missing. Additionally, 4.8% of young people aged 16–17 years reported high DAWBA ratings, while their parents reported low ratings. Overall, 19.9% of CYP and 23.4% of parents reported high DAWBA ratings for BDD.

**Table 1:**
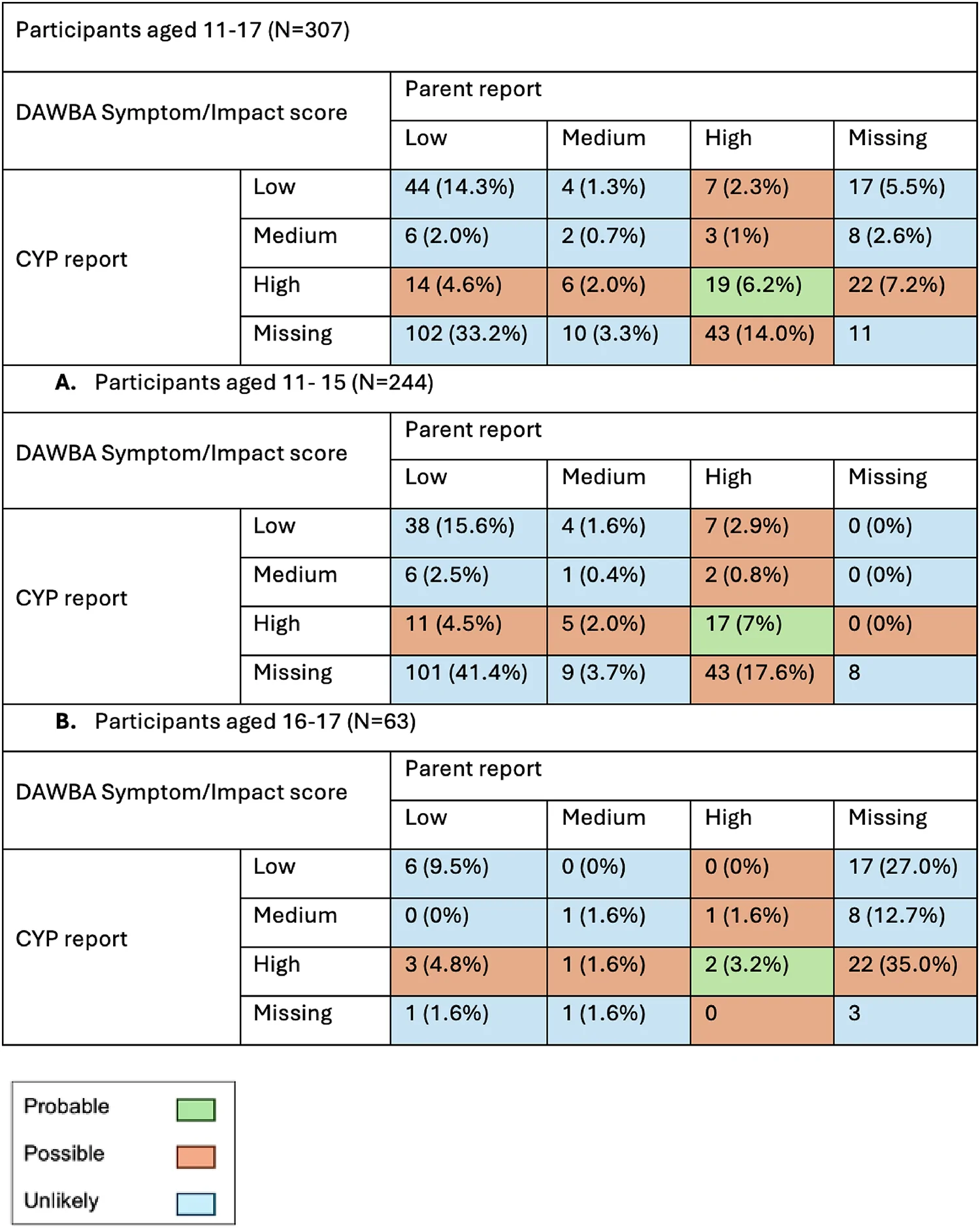
Algorithm-generated diagnostic prediction for BDD based on DAWBA symptom and impact ratings by CYP and/or parents for the total sample, and separately by age groups 11–15 and 16–17 years (data missing for 11 CYP)

^a^ DAWBA symptom and impact ratings by CYP and/or parents are shown for the total participant sample (aged 11–17 years), followed by subgroup of participants aged 11–15 (part A) and 16–17 years (part B)

### Baseline characteristics

**Table 2:** Baseline characteristics of participants aged 11–17 years by BDD likelihood category

	Likelihood of BDD		
	Unlikely (*N* = 193)	Possible (*N* = 95)	Probable (*N* = 19)
**Age**, mean (SD)	13.6 (1.8)	14.3 (1.7)	13.5 (1.3)
**Sex**, female (%)	117 (60.6%)	76 (80.0%)	16 (84.2%)
**Ethnicity**, White, n (%)	162 (83.9%)	82 (86.3%)	17 (89.5%)
**Index of multiple deprivation quintile**, n (%) 1st quintile (most deprived) 2nd quintile 3rd quintile 4th quintile 5th quintile (least deprived)			
	24 (12.4%)	16 (16.8%)	4 (21.1%)
	36 (18.7%)	15 (15.8%)	0 (0.0%)
	49 (25.4%)	17 (17.9%)	3 (15.8%)
	39 (20.2%)	19 (20.0%)	7 (36.8%)
	44 (22.8%)	28 (29.5%)	5 (26.3%)
**Previous CAMHS referral**, n (%)	70 (36.3%)	38 (40.0%)	4 (21.1%)
**At least 1 other emotional disorder domain scoring very high**, n (%)	122 (63.2%)	82 (86.3%)	19 (100.0%)

The demographic characteristics of CYP are summarised in Table 2. Female prevalence was higher across all categories, particularly in the possible and probable BDD categories. In terms of socio-economic status, distribution of deprivation quintiles varied.

Over a third of CYP with possible or unlikely BDD had prior CAMHS referrals compared to only a fifth of those with probable BDD. There was a pattern whereby a higher percentage of individuals scored very high in at least one emotional domain on the DAWBA moving from 66% (unlikely category) to 100% (probable category) (Table 2).

**Fig. 1:**
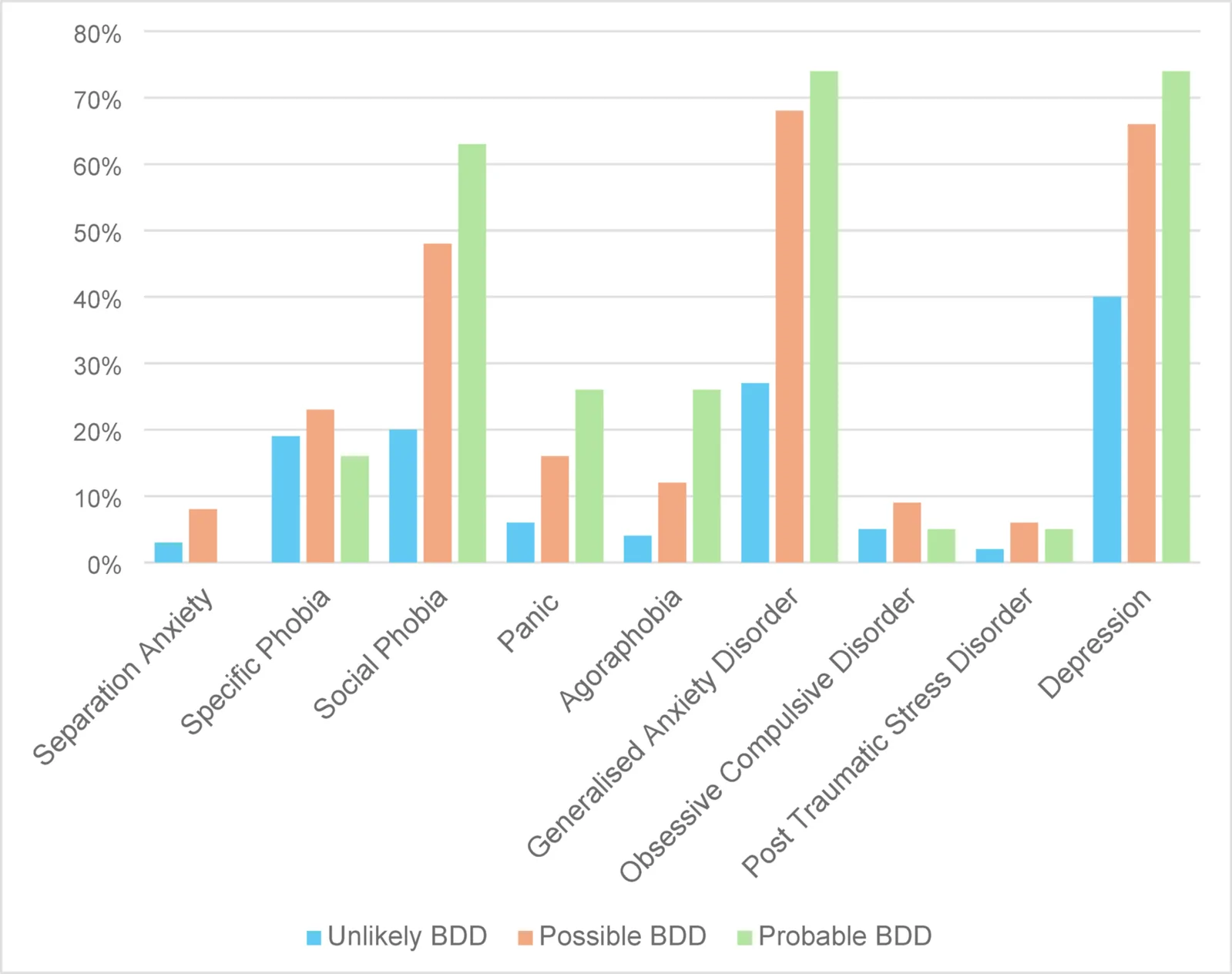
Percentage of CYP for whom algorithm-derived diagnostic prediction shows a very high likelihood of meeting diagnostic criteria for another emotional disorder, by BDD category, based on CYP and/or parent DAWBA responses

CYP with probable BDD were more likely to score very high for the depression, generalised anxiety disorder, social phobia, panic, and agoraphobia domains on the DAWBA, than those with possible or unlikely BDD (Fig. 1). However, this was not the case for OCD, which was relatively uncommon across all BDD categories. Approximately three quarters of CYP with probable BDD scored very high for generalised anxiety disorder and depression, and two thirds for social phobia.

### 12-month outcomes

Overall, around half of the index referrals were accepted into CAMHS across all BDD categories by 12 months (Table 3). When considering any referral to CAMHS over 12 months, acceptance was around 65%. Less than one fifth of CYP received a recorded diagnosis of an emotional disorder by a clinician within 12 months across all categories. No CYP received a recorded diagnosis of BDD by a clinician. Amongst those with probable BDD according to the DAWBA, two young people had a confirmed diagnosis of an emotional disorder at 12 months. Their clinical diagnoses included depression, OCD, generalised anxiety and social anxiety disorder. CAMHS offered treatment/intervention within 12 months to around half of CYP across all BDD categories.

**Table 3:** Service-related outcomes of CYP aged 11–17 years by BDD category based on their healthcare records within 12 months of referral

	Likelihood of BDD		
	Unlikely *N* = 193	Possible *N* = 95	Probable *N* = 19
Acceptance of index referral	98 (50.8%)	47 (49.5%)	9 (47.4%)
Acceptance of any referral	119 (61.7%)	66 (69.5%)	12 (63.2%)
Recorded diagnosis of an emotional disorder by a clinician	27 (14.0%)	17 (17.9%)	2 (10.5%)
Any treatment/intervention offered	83 (43.0%)	54 (56.8%)	9 (47.4%)
Any treatment/intervention started	49 (25.4%)	35 (36.8%)	8 (42.1%)

Table reports n (%)

**Fig. 2:**
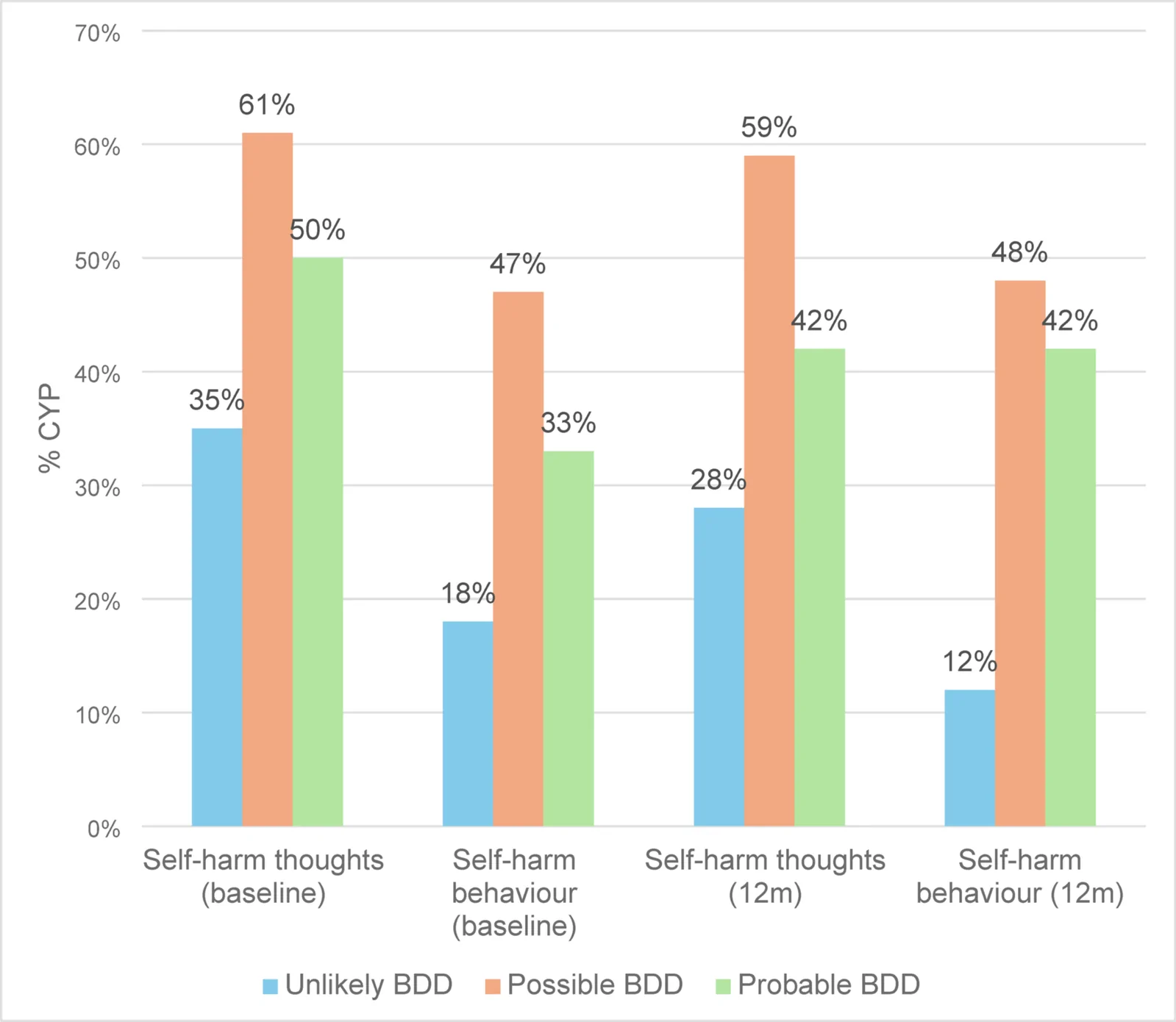
Percentage of CYP reporting self-harm thoughts and self-harm behaviour by BDD category. Percentages are based on available data for each group. N indicates the number of participants with available data at each time point. At baseline, *N* = 18, 64 and 113 for the unlikely, possible and probable BDD categories; at 12 months, *N* = 12, 44 and 68 respectively. Missing data reflect participants lost to follow-up or incomplete responses

CYP in the possible BDD category were more likely to report self-harm thoughts and behaviours than those in the unlikely and probable categories (Fig. 2). Around half of CYP with probable BDD reported self-harm thoughts and a third of them reported self-harm behaviours. At 12 months, compared with baseline, self-harm behaviours were more likely to be reported by those with probable BDD.

Online resource 3 shows the RCADS scores by BDD category at baseline and at 12 months, as reported by CYP and parents separately. CYP with probable BDD reported higher total anxiety scores (68.9 (SD 19.3)) than those in the unlikely category (54.0 (SD 19.3)). Total anxiety scores were lower at 12 months compared to baseline across all BDD categories, with scores in the probable BDD category still being the highest (61.2 (SD 20.8)). When considering CYP-reported RCADS subscales individually, differences between probable and unlikely categories were most prominent in the social phobia, panic disorder and depression subscales. Smaller differences were seen in the generalised anxiety and OCD subscales, with almost no differences between categories in the separation anxiety subscale. For parent-reports (Online Resource 3), differences between BDD categories were smaller but still showing a consistent gradient pattern of higher scores with increasing likelihood of BDD. Similar to CYP reports, parent-reported RCADS scores at 12 months were lower than at baseline, for all subscales.

## Discussion

This study was guided by the need to better understand the characteristics of clinically referred young people that are likely to have BDD, a condition that is under-detected and under-treated [29, 30]. It focused on a clinical sample of CYP with emotional difficulties who were referred to CAMHS. Around 6% of the sample scored very high on appearance pre-occupation based on both self- and parent- reports, indicating an increased likelihood of having BDD. This is likely to be a conservative estimate since the design of the study meant that only one third of the sample had both self- and parent-reported data available. It is notable that 31% of the sample were classified as having possible BDD, as indicated by a high DAWBA rating according to either self-or parent-reports. Nevertheless, our estimate of 6% aligns with a recent meta-analysis in adults showing that 7.4% of patients in outpatient psychiatric settings have BDD [14]. As might be expected in a clinically referred sample, this is substantially higher than the prevalence of 1% in community samples of CYP in the UK [17, 18], although it represents a likelihood prediction rather than a confirmed diagnosis.

In line with previous findings [17, 18], BDD appeared to be more common in females. In our sample, living in a less deprived area was more common amongst CYP with an increased likelihood of BDD than those unlikely to have the condition, although previous findings on the relationship between socio-economic status and BDD have been mixed [31, 32].

CYP with possible or probable BDD were more likely than those in the unlikely category to meet diagnostic criteria for other emotional disorders, consistent with existing literature showing comorbidity is common in affected patients [5, 33]. Given the well-established link between number of comorbidities and functional impairment, this finding highlights the need for early identification and treatment to reduce long-term impairments [34]. Given that all CYP included in this study had been referred to CAMHS and had emotional difficulties, our findings may overestimate the presence of comorbid emotional disorders within general BDD samples. Interestingly, CYP in the possible or probable BDD categories did not show an increased likelihood of also meeting criteria for OCD based on their DAWBA responses, despite the shared conceptualisation of both conditions as obsessive compulsive spectrum disorders. One possible explanation could be that the onset of OCD may be later in individuals with BDD, leading to a greater likelihood of co-occurrence in adulthood than adolescence. However, OCD was less frequent in our sample compared to other comorbidities, making it difficult to draw conclusions. In contrast, CYP with increased likelihood of having BDD were likely to meet the diagnostic criteria for depressive disorder as well as anxiety disorders, specifically generalised anxiety, social phobia, agoraphobia, and panic disorder. Three quarters of CYP with probable BDD, or their parents, reported very high scores for depression and generalised anxiety disorder, showing that they were the most common co-existing conditions in our sample, a finding consistent with previous evidence [5, 18, 30, 35, 36]. This pattern of an increasing likelihood of comorbidity alongside an increasing likelihood of BDD could indicate that BDD may serve as a marker of severity of mental health difficulties for CYP. However, given BDD is overall under-detected, common emotional disorders such as depression or anxiety could also reflect an increased risk of underlying BDD. Further research into the relationships between BDD and other mental health conditions is needed.

In England, the number of CYP seen by CAMHS has grown significantly in the last few years, with almost 950,000 CYP having active referrals in 2022–2023 [37, 38]. The proportion of CYP with previous referrals was lower for CYP with probable BDD (21%) compared to those unlikely to have BDD (36%), possibly due to the identification of more severely affected individuals in the first contact with CAMHS. Delays in seeking help among affected CYP due to the hidden nature of BDD could also lead to less referrals overall for those probably having the condition. Approximately half of the referrals across all BDD categories were accepted. The proportion of CYP receiving a clinician-made diagnosis of any emotional disorder was low (11–18%) irrespective of the likelihood of BDD. No diagnoses of BDD were made and it is not known to what extent BDD was explored and ruled out in CAMHS assessments. BDD is often not considered as a differential diagnosis in routine assessments for young people presenting with non-body image related concerns, despite most people with BDD first accessing services with other concerns, such as mood or anxiety difficulties [38]. The findings may also reflect the overall low rates of diagnoses for CYP with mental health difficulties by UK CAMHS professionals, a wider phenomenon observed in CAMHS that may further complicate the experience of CYP and their families [39]– [40]. Despite this, treatment/intervention was offered to approximately half of CYP referred, irrespective of BDD likelihood. The type or aim of treatment/intervention was not specified and so treatment/intervention may have been offered for difficulties other than BDD-related symptoms for many CYP, especially in the absence of a recorded diagnosis of BDD by a clinician. However, treatment/intervention was started within 12 months of referral in more CYP with probable BDD than those unlikely to have BDD. This may be due to prioritisation of certain CYP based on their overall presentation, severity of symptoms or risks, variations in treatment/intervention availability or young person/family preferences.

In line with previous findings, self-harm was more likely to be reported by CYP with possible or probable BDD compared to those unlikely have BDD [5, 11, 24]. This has important implications as it shows a heightened risk within an already vulnerable group of young people with emotional difficulties. Although the proportion of CYP reporting self-harm was lower or similar over time in those at lower risk of BDD, the proportion of CYP with probable BDD reporting self-harm was higher over 12 months. This could indicate an important risk in this subgroup with reduced likelihood of spontaneous improvement, and potentially poorer prognosis, although more research is needed.

### Strengths and limitations

A strength of this study is its use of a representative, clinically referred sample of CYP with emotional difficulties from a large multi-centre trial, reducing site-specific bias. Notably, there are very few studies looking at BDD in a routine, generic CAMHS referred sample. We aimed to incorporate insights from both CYP and their parent as far as possible to assess the likelihood of BDD, with the DAWBA covering both symptomatology and impairment domains. However, it is important to view the findings in the context of the study’s limitations. Only participants who completed the BDD section of the DAWBA were included in the analysis, which could introduce a selection bias. However, a comparison of baseline characteristics and outcomes between those who completed this section and those who did not revealed no major differences. Due to methodological restrictions of the underlying trial, complete responses from both informants were not available for all participants, potentially affecting the prediction of BDD risk for those with incomplete data. Especially in cases of parent-only reports in the 11–15 year age group, there may be underreporting of BDD-related difficulties as these are often hidden and not shared by CYP for a long time [23, 41]. In the 16–17 years age group, around 40% of CYP reported high DAWBA ratings but were allocated to the possible likelihood category due to either parent reports missing (35%) or parents reporting low (5%) ratings, which could result in an underestimate. Additionally, responses of CYP with BDD may have been influenced by delusionality or impaired insight, which often characterise individuals with BDD [42], potentially affecting the accuracy of self-reports and subsequently the DAWBA predictions. However, in previous research, 97% of young people with BDD endorse the items on appearance preoccupation on the DAWBA [22], indicating most respond positively regardless of insight. RCADS scores were analysed using complete case data only increasing reliability of longitudinal comparisons. Although the DAWBA is a validated tool including an updated section on BDD, the computerised algorithm used to estimate BDD likelihood in this paper has not been previously validated [18]. Therefore, in the absence of adjacent clinical rating, the stratification in likelihood categories should be interpreted with caution. The high levels of comorbid emotional disorders were also based on DAWBA likelihood predictions, not clinician-made diagnoses. The observed level of comorbidity with other emotional disorders could have been partly influenced by referral bias, as CYP in our sample were from a clinically referred sample to CAMHS and are likely to present with more complex symptoms than CYP in the community. Another limitation is the lack of consideration for eating disorders, which may be important comorbid disorders or differential diagnoses in CYP with likely BDD. Although the BDD section of the DAWBA is specific to the diagnostic criteria for BDD (covering worries about specific body features rather than weight or shape concerns) [18], the presence of eating disorders could not be assessed as the corresponding DAWBA section was not administered in the underlying trial. Regarding self-harm, the restricted response options for CYP may have limited our understanding of its frequency and severity and the exclusion of urgent referrals could have led to an underestimate of the self-harm risk. Lastly, the findings are only reliant on descriptive statistics as the nature of the data did not allow for an inferential analysis, therefore presenting observations in the sample but preventing causal attributions.

### Clinical implications and future directions

These findings have several implications for clinical practice. The nature of BDD can make diagnosis particularly challenging, leading to delays in identification [38, 41]. Adolescents often worry about their appearance, making it difficult to delineate where typical concerns end and mild BDD begins, and the diagnosis is largely based on the extent of the impact on daily functioning and level of distress. The reluctance of affected people to seek help further complicates diagnosis and treatment outcomes. Even when seeking professional help, CYP are unlikely to disclose appearance concerns unless directly asked [43]. In clinical practice, individuals with BDD often seek help for other emotional symptoms rather than core appearance concerns, which can make identification of BDD symptoms more challenging [10]. Additionally, limited awareness among CAMHS clinicians and potentially limited confidence in knowing how to assess CYP for BDD, are likely exacerbating this [10]. This highlights an urgent need to improve mental health practitioners’ knowledge on BDD [44]. Routinely asking CYP referred to CAMHS about BDD can help improve identification. Clinicians would likely need to ask directly about appearance preoccupation in their consultations, as many CYP may feel embarrassed to bring this up and parents may not be aware of the extent of the difficulties. Incorporating diagnostic tools in clinical practice could help detection in high-risk populations, such as CYP with probable mood or anxiety disorders given high levels of comorbidity. Future research could focus on establishing the prevalence of BDD in clinical settings such as community CAMHS, and on examining perceived barriers to symptom disclosure and help-seeking. Further work is also needed to investigate the role of improving professional awareness and training to enhance identification in clinical settings.

## Conclusion

Young people with emotional difficulties referred to CAMHS are at risk of having BDD but the condition appears to be under-recognised and underdiagnosed in CAMHS. BDD commonly co-occurs with mood and anxiety disorders, and self-harm thoughts and behaviours. However, the likelihood of BDD does not appear to influence acceptance of referrals by CAMHS or treatments/interventions offered. Improvement of its detection and treatment is paramount to improve outcomes for young people with this condition.

## Supplementary Information

Below is the link to the electronic supplementary material.

Supplementary Material 1

Supplementary Material 2

Supplementary Material 3

## Data Availability

Anonymised trial data may be shared with researchers external to the trial research team in accordance with the NCTU’s data sharing procedure. The datasets containing individual participant data analysed during the STADIA trial will be available upon request from the NCTU (ctu@nottingham.ac.uk) a minimum of 6 months after publication of the NIHR threaded publication. Access to the data will be subject to review of a data sharing and use request by a committee including the Chief Investigator and Sponsor, and should reflect a proposed collaboration with the STADIA trial team, and will only be granted upon receipt of a data sharing and use agreement. Any data shared will be anonymised which may impact on the reproducibility of published analyses.
