# Supplementary material for: Characteristics and 12-month outcomes of clinically-referred children and young people at risk of body dysmorphic disorder
Source: Soc Psychiatry Psychiatr Epidemiol. 2025 Dec 22;61(10):2047–58. doi: 10.1007/s00127-025-03023-x (PMC13585858; doi:10.1007/s00127-025-03023-x)
Supplement: Supplementary file 1 [file 127_2025_3023_MOESM1_ESM.docx]

Characteristics and 12-month outcomes of clinically-referred children and young people at risk of Body Dysmorphic Disorder

Eleni Frisira^1^, Sue Fen Tan^1^, Chris Partlett^2^, Grace Holt^2^, Georgina Krebs^3^, Argyris Stringaris^4^, Tamsin Marshall^5^, Kapil Sayal^1^

Affiliations

^1^Institute of Mental Health, School of Medicine, Mental Health and Clinical Neurosciences, University of Nottingham, Nottingham, NG7 2TU, United Kingdom.

^2^Nottingham Clinical Trials Unit, University of Nottingham, Building 42, Applied Health Research, Nottingham NG7 2RD, United Kingdom.

^3^Research Department of Clinical, Educational and Health Psychology, 1-19 Torrington Place, University College London, London, WC1E 7HB.

^4^Faculty of Brain Sciences, University College London, London, United Kingdom; National and Kapodistrian University of Athens, Athens, Greece.

^5^Berkshire Healthcare NHS Foundation Trust, Bracknell, UK.

Corresponding Author: eleni.frisira@nottingham.ac.uk

**Online Resource 1: Participant flow diagram from the STADIA trial for participant inclusion in current study.**
