# Supplementary material for: Characteristics and 12-month outcomes of clinically-referred children and young people at risk of body dysmorphic disorder
Source: Soc Psychiatry Psychiatr Epidemiol. 2025 Dec 22;61(10):2047–58. doi: 10.1007/s00127-025-03023-x (PMC13585858; doi:10.1007/s00127-025-03023-x)
Supplement: Supplementary file 2 [file 127_2025_3023_MOESM2_ESM.docx]

Characteristics and 12-month outcomes of clinically-referred children and young people at risk of Body Dysmorphic Disorder

Eleni Frisira^1^, Sue Fen Tan^1^, Chris Partlett^2^, Grace Holt^2^, Georgina Krebs^3^, Argyris Stringaris^4^, Tamsin Marshall^5^, Kapil Sayal^1^

Affiliations

^1^Institute of Mental Health, School of Medicine, Mental Health and Clinical Neurosciences, University of Nottingham, Nottingham, NG7 2TU, United Kingdom.

^2^Nottingham Clinical Trials Unit, University of Nottingham, Building 42, Applied Health Research, Nottingham NG7 2RD, United Kingdom.

^3^Research Department of Clinical, Educational and Health Psychology, 1-19 Torrington Place, University College London, London, WC1E 7HB.

^4^Faculty of Brain Sciences, University College London, London, United Kingdom; National and Kapodistrian University of Athens, Athens, Greece.

^5^Berkshire Healthcare NHS Foundation Trust, Bracknell, UK.

Corresponding Author: eleni.frisira@nottingham.ac.uk

**Supplementary Material 2: Cross-tabulation of DAWBA Algorithm scores for Symptom and Impact Ratings in the “Worrying About Physical Appearance” Section.** The DAWBA scores are assigned by the computerised algorithm based on informant responses, ranging from “– –”to “+++”. Each level corresponds to an approximate epidemiological prevalence estimate in British child and adolescent samples. In the present study, severity levels were defined as:
**High** = (+++/+++),
**Medium** = (++/+++, +++/++ or ++/++),
**Low** = all other combinations, and
**Missing** = both symptom and impact scores missing or not applicable.

| **Parent symptom** | **Parent impact** | | | | | | | **TOTAL** |
| --- | --- | --- | --- | --- | --- | --- | --- | --- |
|  | - - | - | -/+ | + | + + | + + + | missing or n/a |  |
| - - | 0 | 0 | 0 | 0 | 0 | 0 | 0 | **0** |
| - | 0 | 0 | 0 | 0 | 0 | 0 | 87 | **87** |
| -/+ | 0 | 0 | 0 | 0 | 0 | 0 | 0 | **0** |
| + | 0 | 9 | 0 | 0 | 3 | 9 | 42 | **63** |
| + + | 0 | 0 | 0 | 0 | 1 | 2 | 5 | **8** |
| + + + | 0 | 11 | 0 | 0 | 19 | 72 | 0 | **102** |
| missing or n/a | 0 | 0 | 0 | 0 | 0 | 0 | 58 | **58** |
| **TOTAL** | **0** | **20** | **0** | **0** | **23** | **83** | **192** | **318** |

| **YP symptom** | **YP impact** | | | | | | | **TOTAL** |
| --- | --- | --- | --- | --- | --- | --- | --- | --- |
|  | - - | - | -/+ | + | + + | + + + | missing or n/a |  |
| - - | 0 | 0 | 0 | 0 | 0 | 0 | 0 | **0** |
| - | 0 | 0 | 0 | 0 | 0 | 0 | 22 | **22** |
| -/+ | 0 | 0 | 0 | 0 | 0 | 0 | 0 | **0** |
| + | 0 | 8 | 0 | 0 | 1 | 1 | 22 | **32** |
| + + | 0 | 3 | 0 | 0 | 2 | 0 | 2 | **7** |
| + + + | 0 | 13 | 0 | 0 | 17 | 61 | 0 | **91** |
| missing or n/a | 0 | 0 | 0 | 0 | 0 | 0 | 166 | **166** |
| **TOTAL** | **0** | **24** | **0** | **0** | **20** | **62** | **212** | **318** |
