# Supplementary material for: Characteristics and 12-month outcomes of clinically-referred children and young people at risk of body dysmorphic disorder
Source: Soc Psychiatry Psychiatr Epidemiol. 2025 Dec 22;61(10):2047–58. doi: 10.1007/s00127-025-03023-x (PMC13585858; doi:10.1007/s00127-025-03023-x)
Supplement: Supplementary file 3 [file 127_2025_3023_MOESM3_ESM.docx]

Characteristics and 12-month outcomes of clinically-referred children and young people at risk of Body Dysmorphic Disorder

Eleni Frisira^1^, Sue Fen Tan^1^, Chris Partlett^2^, Grace Holt^2^, Georgina Krebs^3^, Argyris Stringaris^4^, Tamsin Marshall^5^, Kapil Sayal^1^

Affiliations

^1^Institute of Mental Health, School of Medicine, Mental Health and Clinical Neurosciences, University of Nottingham, Nottingham, NG7 2TU, United Kingdom.

^2^Nottingham Clinical Trials Unit, University of Nottingham, Building 42, Applied Health Research, Nottingham NG7 2RD, United Kingdom.

^3^Research Department of Clinical, Educational and Health Psychology, 1-19 Torrington Place, University College London, London, WC1E 7HB.

^4^Faculty of Brain Sciences, University College London, London, United Kingdom; National and Kapodistrian University of Athens, Athens, Greece.

^5^Berkshire Healthcare NHS Foundation Trust, Bracknell, UK.

Corresponding Author: eleni.frisira@nottingham.ac.uk

**Online Resource 2: Revised Children Anxiety and Depression Scale (RCADS) scores by BDD category, as reported by CYP (A) and parents (B). Data presented are complete cases.**

A. **RCADS (CYP report) by BDD categories**

|  | Likelihood of BDD | | |
| --- | --- | --- | --- |
|  | Unlikely | Possible | Probable |
|  | N=66 | N=41 | N=11 |
| Social phobia subscale | | | |
| Baseline | 17.4 (5.8) | 19.3 (5.8) | 22.1 (3.5) |
| 12 months | 15.8 (7.1) | 18.7 (6.8) | 20.5 (5.8) |
| Panic disorder | | | |
| Baseline | 11.7 (6.6) | 14.6 (6.8) | 16.1 (7.4) |
| 12 months | 9.9 (7.1) | 13.7 (7.3) | 14.8 (4.9) |
| Depression subscale | | | |
| Baseline | 16.2 (5.7) | 19.8 (6.2) | 20.9 (5.9) |
| 12 months | 14.2 (7.3) | 18.5 (6.8) | 16.9 (5.4) |
| Separation Anxiety subscale | | | |
| Baseline | 8.1 (5.0) | 8.2 (4.5) | 8.5 (4.3) |
| 12 months | 6.8 (4.7) | 8.2 (5.1) | 8.4 (5.0) |
| Generalised Anxiety | | | |
| Baseline | 9.6 (4.5) | 11.7 (4.7) | 12.5 (4.7) |
| 12 months | 7.7 (4.8) | 10.7 (4.7) | 9.9 (4.7) |
| OCD | | | |
| Baseline | 7.3 (3.8) | 8.0 (4.5) | 9.8 (4.1) |
| 12 months | 5.5 (4.7) | 8.3 (4.6) | 7.6 (4.5) |
| Total anxiety score | | | |
| Baseline | 54.0 (19.3) | 61.7 (22.5) | 68.9 (19.3) |
| 12 months | 45.6 (23.3) | 59.6 (24.5) | 61.2 (20.8) |
| Total Internalizing Scale | | | |
| Baseline | 70.2 (23.1) | 81.5 (27.4) | 89.8 (24.4) |
| 12 months | 59.7 (29.5) | 78.1 (30.3) | 78.1 (25.4) |
| Table reports Mean (SD) unless otherwise specified | | | |

| B. **RCADS (parent report) by BDD categories** | | | |
| --- | --- | --- | --- |
|  | Likelihood of BDD | | |
|  | Unlikely | Possible | Probable |
|  | N=113 | N=50 | N=10 |
| Social phobia subscale | | | |
| Baseline | 15.2 (6.4) | 17.9 (5.1) | 19.5 (4.7) |
| 12 months | 13.5 (6.7) | 17.0 (6.1) | 17.4 (6.3) |
| Panic disorder | | | |
| Baseline | 7.0 (4.9) | 9.9 (6.3) | 7.9 (4.5) |
| 12 months | 6.1 (5.0) | 9.4 (6.5) | 8.6 (5.4) |
| Depression subscale | | | |
| Baseline | 13.3 (5.6) | 16.3 (6.1) | 14.3 (4.2) |
| 12 months | 10.1 (6.0) | 14.5 (6.9) | 11.7 (5.9) |
| Separation Anxiety subscale | | | |
| Baseline | 7.7 (5.3) | 8.2 (4.9) | 5.8 (3.8) |
| 12 months | 6.2 (4.8) | 8.0 (4.9) | 6.2 (2.5) |
| Generalised Anxiety | | | |
| Baseline | 8.0 (4.0) | 9.3 (3.9) | 10.1 (4.7) |
| 12 months | 6.2 (3.9) | 8.5 (4.4) | 8.7 (4.2) |
| OCD | | | |
| Baseline | 4.9 (4.0) | 4.7 (3.5) | 5.2 (3.3) |
| 12 months | 3.6 (3.7) | 5.2 (4.1) | 4.1 (4.0) |
| Total anxiety score | | | |
| Baseline | 42.9 (18.9) | 50.1 (19.2) | 48.5 (15.3) |
| 12 months | 35.5 (19.5) | 48.1 (22.0) | 45.0 (17.2) |
| Total Internalizing Scale | | | |
| Baseline | 56.2 (21.7) | 66.4 (23.5) | 62.8 (17.5) |
| 12 months | 45.6 (23.8) | 62.7 (27.7) | 56.7 (22.1) |
| Table reports Mean (SD) unless otherwise specified | | | |
